# Distribution, characterization, and antibiotic resistance of hypervirulent *Klebsiella pneumoniae* isolates in a Chinese population with asymptomatic bacteriuria

**DOI:** 10.1186/s12866-021-02413-w

**Published:** 2022-01-18

**Authors:** Jun Li, Yanbing Li, Mengli Tang, Fengjun Xia, Changhang Min, Yongmei Hu, Haichen Wang, Jingyi Zhang, Mingxiang Zou

**Affiliations:** 1 Department of Clinical Laboratory, Xiangya Hospital, Central South University, Changsha, 410008 Hunan China; 2 National Clinical Research Center for Geriatric Disorders, Xiangya Hospital, Central South University, Changsha, 410008 Hunan China

**Keywords:** Hypervirulent *K. pneumoniae*, Asymptomatic bacteriuria, Antimicrobial susceptibility, Virulence-associated genes, Biofilm formation, Whole-genome sequencing (WGS)

## Abstract

**Background:**

Asymptomatic bacteriuria (ASB) frequently occurs among all ages and may develop into urinary tract infections (UTIs). Hypervirulent *Klebsiella pneumoniae* (hvKP) has become a new threat to human health. In our study, we aimed to investigate the epidemiological characteristics of hvKP in population with ASB.

**Results:**

A total of 61 *K. pneumoniae* isolates were collected from 7530 urine samples between October and December 2020. The strains were sensitive to most of the antimicrobial agents tested, but a polymyxin resistant strain was found (MIC>16 μg/mL). Three serotypes were detected, including K1 (16.4%, 10/61), K5 (1.6%, 1/61) and K57 (3.2%, 2/61). Four strains (KPNY9, KPNY31, KPNY40, and KPNY42) carried a combination of two or more hypervirulent markers (*peg-344*, *iroB*, *iucA*, _*p*_*rmpA*, and _*p*_*rmpA2*), and their survival rates after *Galleria mellonella* infection were lower than those of the other strains (40.0 *vs*. 70.0%), suggesting that they were hvKP. These hvKP strains with lower biofilm forming ability than classical *K. pneumoniae* (0.2625 ± 0.0579 *vs*. 0.6686 ± 0.0661, *P* = 0.033) were identified as belonging to K2-ST65, K2-ST86, K57-ST592, and K2-ST5559 (a new ST type). KPNY31 (ST5559) shared a close genetic relationship with KPNY42 (ST86) and other ST86 isolates, which have been detected in both nosocomial and community-acquired infections.

**Conclusions:**

The hvKP with relatively weak biofilm formation was detected in a population with ASB, which was more likely to cause bacteremia and serious consequences. A novel sequence type (ST5559) hvKP derived from ST86 was found. Therefore, hvKP should be monitored in the population with ASB.

## Background

Urinary tract infections (UTIs), which affect 150 million people each year, have emerged as one of the most common bacterial infections that affect people of all ages and genders [1]. The diagnosis requires a combination of relevant signs, symptoms, and a positive urine culture (UC). Positive urine culture without associated signs and symptoms is defined as asymptomatic bacteriuria (ASB) [2]. ASB becomes more common with age, from < 2% in children to up to 50% in elderly residents of long-term care facilities [2].

ASB is frequently caused by the same types of bacteria as in symptomatic UTIs, such as *Escherichia coli*, *Klebsiella pneumoniae*, *Enterococcus faecium*, *Enterococcus faecalis*, and so on, among which *E. coli* and *K. pneumoniae* are mainly predominant [3]. Hypervirulent *K. pneumoniae* (hvKP) is a variant of *K. pneumoniae* (KP), but more pathogenic, easily transmitted, and with a higher mortality rate [4, 5]. It can cause multiple sites of infection, including abdominal disease, thoracic disease, endophthalmitis, central nervous system disease, and genitourinary tract infections [5]. Recently, it has also been detected in UTIs with multidrug resistance and even extensively drug-resistant isolates [6–8]. Worse yet, hvKP infection was more likely to cause bacteremia and serious consequences than classical *K. pneumoniae* (cKP) when it developed into UTIs [9].

However, studies on hvKP in ASB are still rare [10]. Therefore, our study investigated the molecular epidemiological characteristics of hvKP in ASB to provide guidelines for the empirical treatment of UTIs.

## Results

### Characteristics of cKP and hvKP isolates

**Fig. 1:**
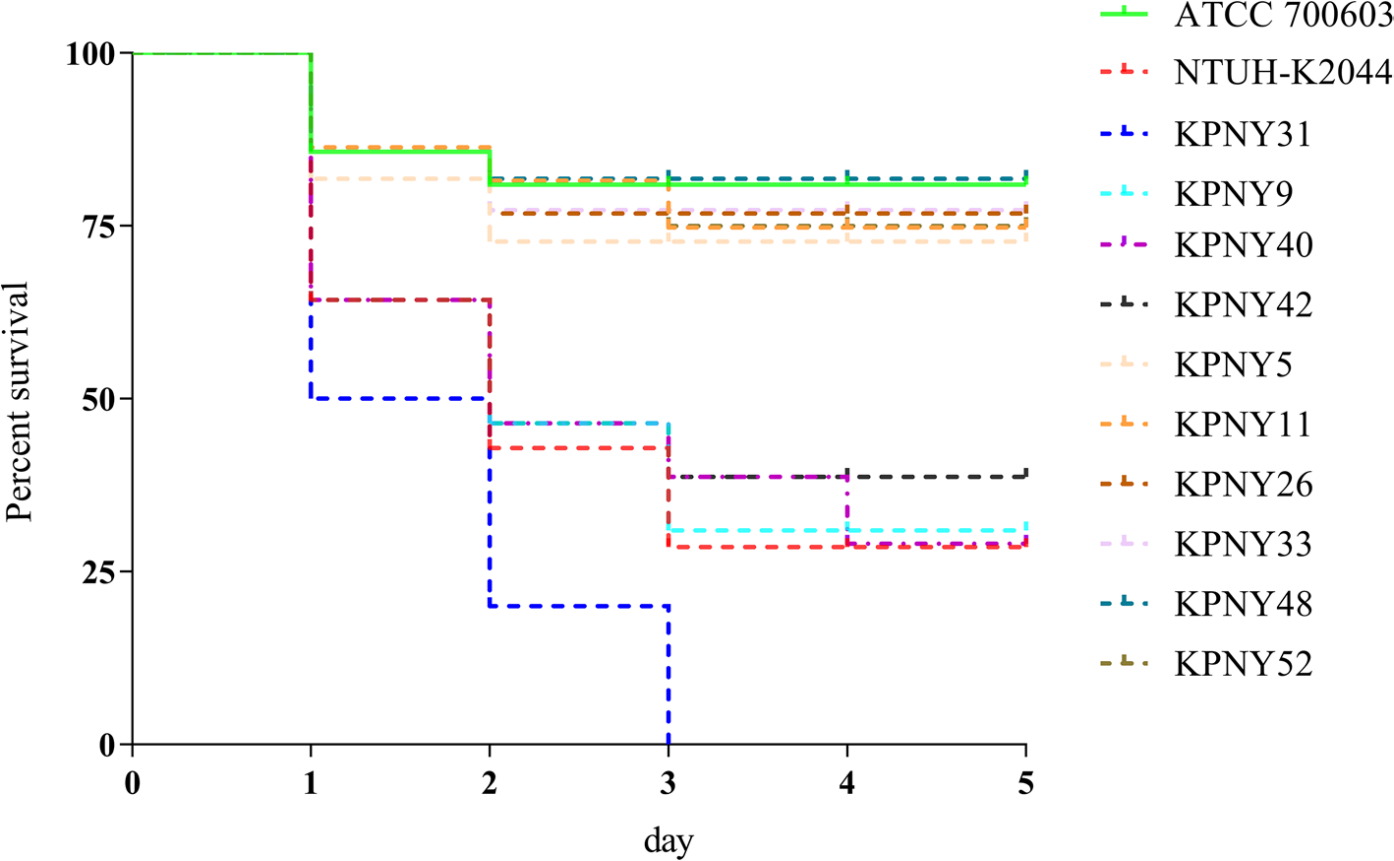
Virulence of KP isolates. The virulence of 1 × 10^6^ CFU of each KP strain (KPNY9, KPNY31, KPNY40, KPNY42, KPNY5, KPNY11, KPNY26, KPNY33, KPNY48, and KPNY52) was investigated using a *G. mellonella* infection model. ATCC 700603 and NTUH-K2044 were used as low and high virulence KP controls, respectively. The four isolates (KPNY9, KPNY31, KPNY40, KPNY42) resulted in significantly lower survival rates than ATCC 700603, suggesting that they were hypervirulent

**Fig. 2:**
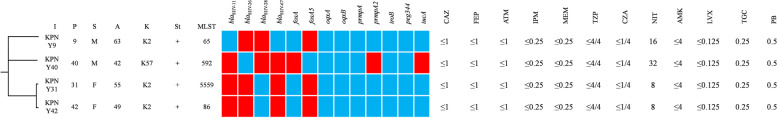
Clinical characteristics, antimicrobial susceptibility, resistance genes, and virulence-associated genes of four hvKP isolates. I, isolate; P, patient; S, sex; A, age; K, K serotype; St, string test; CAZ, ceftazidime; FEP, cefepime; IPM, imipenem; MEM, meropenem; CZA, ceftazidime / avibactam; ATM, aztreonam; TZP, piperacillin / tazobactam; NIT, nitrofurantoin; AMK, amikacin; LVX, levofloxacin; TGC, tigecycline; PB, polymyxin. The red and blue squares indicate the absence and presence of the genes indicated at the top, respectively

A total of 7530 urine samples were collected and 61 *K. pneumoniae* isolates were found from 61 people, 20 men and 41 women, respectively. *Galleria mellonella* has long been used as an infection model for studying bacterial and fungal infections, as well as evaluating the efficacy of new antimicrobial drugs. In our current study, using the *G. mellonella* infection model, we investigated the virulence of the strains and discovered that the survival rates after infection with four strains (KPNY9, KPNY31, KPNY40, and KPNY42) were less than 40.0 percent. In contrast, the other isolates in our study had a survival rate of more than 70.0 percent (Fig. 1). Furthermore, the four strains had hypermucoviscosity, which belonged to K2 or K57, and co-carried two or more hypervirulent markers (*peg-344*, *iroB*, *iucA*, _*p*_*rmpA*, and _*p*_*rmpA2* genes), indicating that they were hvKP (Fig. 2).

There was no significant difference in basic subjects’ demographics between the cKP group and the hvKP group, such as age, sex, white blood cell (WBC) count, and nitrite. The detailed baseline characteristics of the enrolled participants are summarized in Table 1.

**Table 1:** Comparison of clinical characteristics and antimicrobial susceptibility patterns between hvKP and cKP isolates

Clinical characteristic	hvKP (***n*** = 4)	cKP (***n*** = 57)	***P*** value
Basic demographics			
Age	52.25 ± 4.46	42.72 ± 1.715	0.154
Male	2 (50.0%)	18 (31.6%)	0.835
WBC (0)	4 (100.0%)	57 (100.0%)	-
Nitrite (negative)	4 (100.0%)	57 (100.0%)	-
Antimicrobial susceptibility [n (S, %)]			
Ceftazidime	4 (100.0%)	53 (93.0%)	1.000
Cefepime	4 (100.0%)	52 (91.2%)	1.000
Imipenem	4 (100.0%)	57 (100.0%)	-
Meropenem	4 (100.0%)	57 (100.0%)	-
Ceftazidime / avibactam	4 (100.0%)	57 (100.0%)	-
Aztreonam	4 (100.0%)	52 (91.2%)	1.000
Piperacillin / tazobactam	4 (100.0%)	57 (100.0%)	-
Nitrofurantoin	2 (50.0%)	46 (80.7%)	0.196
Amikacin	4 (100.0%)	57 (100.0%)	-
Levofloxacin	4 (100.0%)	53 (93.0%)	1.000
Tigecycline	4 (100.0%)	57 (100.0%)	-
Polymyxin	4 (100.0%)	56 (98.2%)	1.000

*n*, number, *S* sensitive, - not determined

The *bla*_SHV-28_, *fosA5*-like, *oqxA*, and *oqxB* genes were detected in KPNY31 and KPNY42. The *bla*_SHV-11_, *bla*_SHV-67_, *fosA5*-like, *oqxA* and *oqxB* genes were present in KPNY9, while *bla*_SHV-26_, *fosA5*, *oqxA*, and *oqxB* were detected in KPNY40 (Fig. 2)

### Antimicrobial susceptibility of *K. pneumoniae* isolates

We tested the sensitivity of the isolated KP strains to a panel of antimicrobial agents and discovered that all strains were sensitive to the most of agents detected. The sensitivity rates of the 61 KP isolates to ceftazidime (CAZ), cefepime (FEP), aztreonam (ATM), nitrofurantoin (NIT), and levofloxacin (LVX) were 96.7% (59/61), 91.8% (56/61), 95.1% (58/61), 98.4% (60/61), and 98.4% (60/61), respectively; the strains demonstrated 100.0% sensitivity to ceftazidime / avibactam (CZA), piperacillin / tazobactam (TZP), imipenem (IPM), meropenem (MEM), amikacin (AMK), and tigecycline (TGC). One strain was resistant to polymyxin but susceptible to all other antimicrobial agents (except for NIT). There was no significant difference in drug sensitivity between hvKP and cKP isolates in our study (Table 1).

### Virulence factors

Four serotypes were identified among the 61 KP isolates: K1, K2, K5, and K57. In addition, K2 and K57 serotypes were detected in hvKP strains, with a predominance of K2 serotypes (3/4). In 57 cKP isolates, three serotypes were found: K1, K5, and K57, with rates of 17.5, 1.8, and 3.5%, respectively. Four of the hvKP isolates and four of the 57 cKP isolates had hypermucoviscosity. When compared to cKP isolates, hvKP isolates had higher rates of K2 and *prmpA + prmpA2 + iucA + iroB + peg-344*. Furthermore, the hypermucoviscous phenotype was more prevalent in hvKP isolates (Table 2).

**Table 2:** Comparison of virulence factors between hvKP and cKP isolates

Virulence factors	hvKP (***n*** = 4)	cKP (***n*** = 57)	***P*** value
K serotype			
K1	0	10 (17.5%)	1.000
K2	3 (75.0%)	0	**0.000**
K5	0	1 (1.8%)	1.000
K57	1 (25.0%)	2 (3.5%)	0.187
hypermucoviscosity	4 (100.0%)	4 (7.0%)	**0.000**
_*p*_*rmpA* + *iroB* + *peg-344*	1 (25.0%)	0	0.066
_*p*_*rmpA* + _*p*_*rmpA2* + *iucA* + *iroB* + *peg-344*	3 (75.0%)	0	**0.000**

### Biofilm formation

**Fig. 3:**
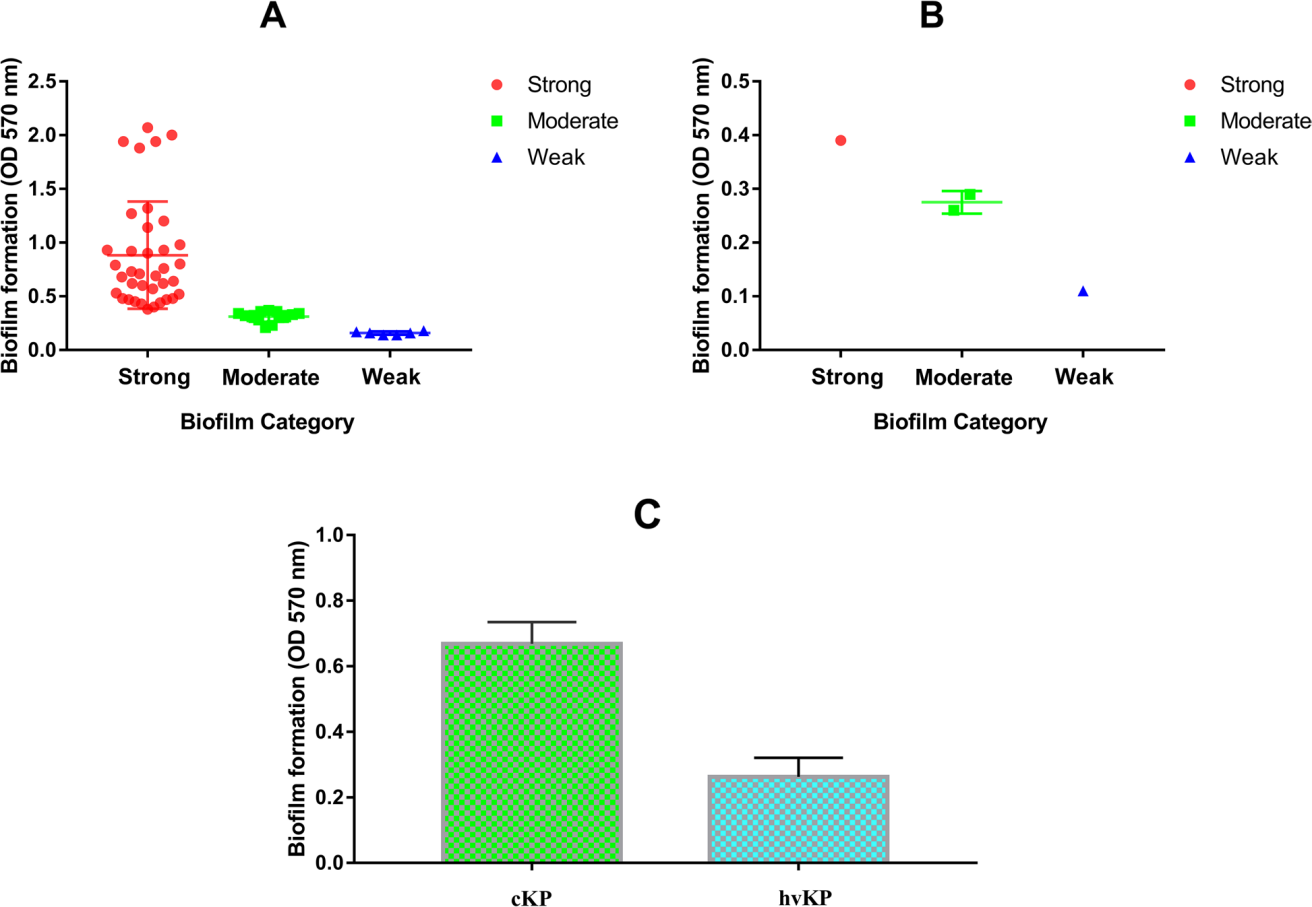
Biofilm formation of hvKP and cKP. Biofilm formation was determined using crystal violet assay in 96-well plates. **A** Quantification and categorization of biofilm formation of cKP isolates. **B** Quantification and categorization of biofilm formation of hvKP isolates. **C** Biofilm formation of hvKP and cKP was compared; there was a significant difference in the biofilm formation between hvKP and cKP (0.2625 ± 0.0579 *vs*. 0.6686 ± 0.0661, *P* = 0.033)

We investigated the biofilm formation of KP isolates and discovered that all KP isolates were biofilm producers, albeit to varying degrees: strong biofilm producers (63.9%, 39/61), medium biofilm producers (24.6%, 15/61), and weak biofilm producers (11.5%, 7/61). In 1, 2, and 1 hvKP isolates, strong biofilm, medium biofilm, and weak biofilm formation were observed, respectively (Fig. 3A). KPNY31 had a moderate ability to form biofilms. There were 38 cKP strains with strong biofilm formation, 13 with medium biofilm formation, and 6 with weak biofilm formation among the cKP strains (Fig. 3B). cKP was more capable of forming biofilms than hvKP (0.6686 ± 0.0661 *vs*. 0.2625 ± 0.0579, *P* = 0.033) (Fig. 3C).

### Phylogenic analysis

The results of sequencing showed that among the four hvKP strains (KPNY9, KPNY31, KPNY40, and KPNY42), KPNY31 and KPNY42 were more closely related, and the remaining two strains were more distantly related (Fig. 2). In addition, MLST analysis revealed that the four hvKP strains were classified as ST65, ST86, ST592, and ST5559 (9-4-2-363-1-1-27, a new ST type). ST5559 hvKP’s resistance pattern, set of resistant genes, and virulence-related genes were identical to those of ST86’s KPNY42 (Fig. 2). BacWGSTdb 2.0 was used to perform phylogenetic analysis on the ST5559 isolate (KPNY31) with the following parameters: SNP threshold of 200, MLST scheme of cgMLST, and MLST threshold of 500. Using the cgMLST strategy, a total of 55 closely related isolates were identified, all of which belonged to ST86.

**Fig. 4:**
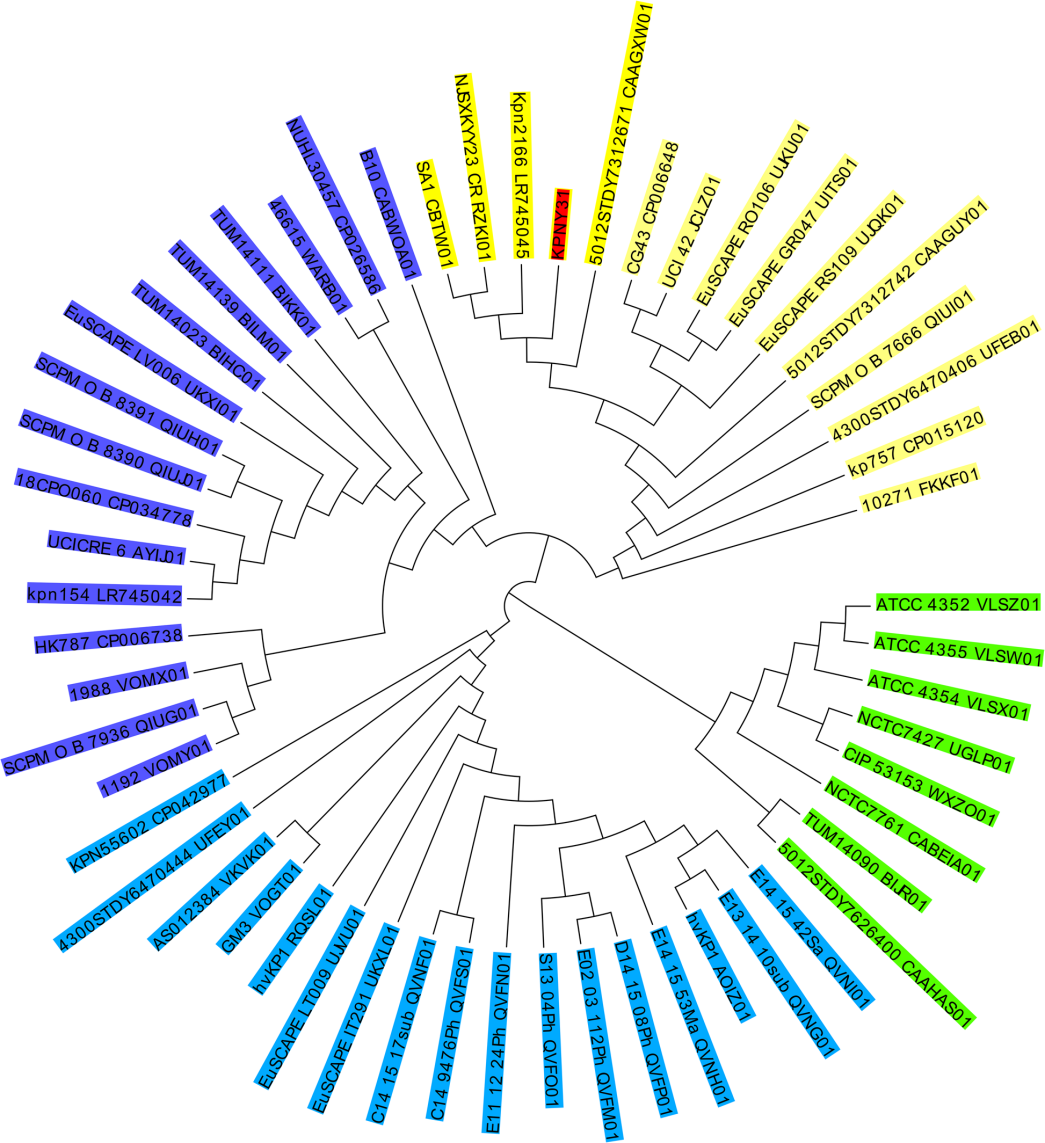
The phylogenetic analysis of ST5559. Phylogenic analysis was performed using BacWGSTdb 2.0 with the following parameters: SNP threshold of 200, MLST scheme with cgMLST and MLST threshold of 500. The isolates were divided into four groups: **A** (yellow), **B** (dark blue), **C** (light blue), and **D** (green). ST5559 belonged to group A

KPNY31 (ST5559) belonged to group A of the isolates, which were divided into four groups (A–D). SA1 (accession number: CBTW01), a hvKP isolated from France, was the most closely related isolate to KPNY31, with 67 different alleles (Fig. 4.).

## Discussions

ASB frequently occurs among all ages with the possibility of developing into UTIs. HvKP has become a new threat to human health and has also been reported in UTIs with multidrug resistance and even extensively drug-resistant isolates. However, the epidemiological characteristics of hvKP in a population with ASB were uncovered. This study examined hvKP isolated from population with ASB at our hospital from October to December 2020. Four hvKP strains were detected from 61 KP isolates, with a rate of 6.6%. The detection rate was lower than that of Zafar et al*.* reported in complicated urinary tract infections in kidney stone patients with 29.5% (18/61), and Taraghian et al*.* reported in urinary tract infections with 10.5% (11/105) [6, 11].

In this study, all 61 KP strains were sensitive to most of the tested antibiotics. It is worth noting that one strain showed resistance to polymyxin. Polymyxin has been used as an important antibiotic in the treatment of multidrug-resistant bacterial infections. However, due to its widespread clinical use, polymyxin-resistant strains are becoming more prevalent because of the plasmid-mediated *mcr* gene, and polymyxin-resistant hvKP strains have emerged [12, 13]. In addition, antibiotics are widely used in animals for disease prevention and treatment, which promotes the emergence of polymyxin-resistant strains [14], and humans can acquire drug-resistant strains through diet and other routes.

We examined virulence-related genes in these strains; eight strains were string test–positive, while four strains were identified as hvKP by *G. mellonella* larvae, indicating that not all string test–positive strains were hvKP, which is consistent with some previous reports [15]. Three K2 strains and one K57 strain were hvKP, while all K1 serotype strains were non-hvKP. Six strains carried two or more virulence-related genes simultaneously. Four of these isolates were identified as hvKP, indicating that isolates carrying multiple virulence genes at the same time considerably enhanced the chance of hvKP strains.

Previous research has linked the K2 serotype of hvKP to a broader range of STs, such as ST25, ST65, ST66, and ST86 [16–19], which have been detected not only in nosocomial infections but also in community-acquired infections [20–23]. These results indicate that these ST types of hvKP may be transmitted both within and outside of hospitals. In our study, two K2 serotype hvKP strains belonging to ST65 and ST86 were detected in a population with ASB, suggesting that ST65 and ST86 hvKP strains could be an important reservoir for UTIs. Another K2 serotype hvKP strain was identified to belong to ST5559 (a new ST type). Notably, the resistance pattern, set of resistant genes, and virulence-related genes of the ST5559 hvKP were identical to KPNY42, which belongs to ST86. Furthermore, compared with other isolates using BacWGSTdb 2.0, the strain had a close genetic relationship with SA1 (accession number: CBTW01) with 67 different alleles (ST86), which was hvKP from Paris, France, illustrating that the strain could have evolved from ST86 but the specific communication mechanism is not precise. In addition to the K2 serotype hvKP, K57 was also detected in our study; this serotype has been reported in other regions of China [24] and Japan [25]. To the best of our knowledge, this is the first study revealing that K57 hvKP belongs to ST592.

Biofilm formation plays an important role in the invasion of KP into bladder epithelial cells as well as the escape of phagocytic phagocytosis in UTIs [26]. Our study showed that all hvKP had biofilm formation ability, and there was no difference in the detection rate of hvKP and cKP. Cubero et al. reported that hvKP could form robust biofilms at the air-liquid interface [27]. However, this finding was inconsistent with the report of Zafar et al., which showed that the biofilm formation ability of hvKP was higher than that of cKP from complicated urinary tract infection in kidney stone patients (94.4 *vs*. 86.0%) [6]. These conflicting results may be related to the different subject groups. All subjects in this study were the population with ASB. Of course, this may also be related to only four hvKP strains detected in this study, and the few strains may not be representative. Although all hvKP isolates in our study produced biofilm, the optical density (OD) value was significantly lower than that of cKP, suggesting that the biofilm-forming ability of hvKP was weaker than that of cKP and these were more likely to cause bacteremia and serious consequences when it developed into UTIs [9].

This study has some limitations. First, the time required to collect strains within several months is relatively brief. Additionally, our study examined a small number of strains. Finally, biomarker profiles and *G. mellonella* larvae were used to determine hvKP isolates rather than a mouse model.

## Conclusions

Although the majority of the KP isolates identified in our study were susceptible to most antibiotics, a polymyxin-resistant strain was found. Notably, hvKP isolates were detected in a population with ASB with lower biofilm formation ability than that of cKP and easily caused systemic infection with serious consequences. This is the first report of ST5559 hvKP, and our results indicate that it has some relationship with ST86 detected in nosocomial infections. Therefore, hvKP strains should be monitored in the population with ASB.

## Methods

### Collection and identification of *K. pneumoniae* isolates

*K. pneumoniae* isolates were collected from urine specimens of population with no symptoms of UTIs at Central South University’s Xiangya Hospital (Changsha, China) from October to December 2020. Xiangya Hospital is a general hospital with 3,500 beds and manages approximately 3 million patients from all over the country every year. Aseptic operation was used to collect 5-10 mL of freshly voided midstream urine with sterile and wide-mouthed plastic bottles with a tight cap. Using calibrated inoculating loop 0.001 mL of uncentrifuged, uniformly mixed, midstream urine samples were aseptically inoculated onto blood agar (Guangzhou Boret Biotechnology Co., Ltd, China). After overnight incubation at 37 °C for 24–48 h colonies were counted to check significant growth. Colony counts yielding bacterial growth of 10^5^/mL urine were regarded as significant for bacteriuria [2]. All isolates were identified using matrix-assisted laser desorption/ionization time-of-flight mass spectrometry (MALDI-TOF MS; Bruker Daltonics GmbH, Bremen, Germany) with *Escherichia coli* ATCC 25922 as a quality control strain (National Center for Clinical Laboratories, Beijing, China). Briefly, one colony from an overnight culture was taken with a disposable loop and spotted onto a metal plate and the spots were then covered with 1 μL of a α-cyano-4-hydroxy-cinnamic acid (HCCA) matrix (Bruker Daltonik GmbH, Bremen, Germany). Then, bacterial samples on the microplate were analyzed with MALDI-TOF MS. Finally, MALDI Biotyper® (Bruker Daltonik GmbH, Bremen, Germany) software was used to classify the isolate at the genus and species level.

### Identification of hvKP isolates

The string test was carried out to characterize the hypermucoviscous phenotype [28]. HvKP isolates were screened for the presence of _*p*_*rmpA*, _*p*_*rmpA2*, *iroB*, *peg344*, and *iucA* or any combination of these genes as previously described [15]. The virulence of all the collected isolates was evaluated using an infection model of *G. mellonella* larvae (Tianjin Huiyude Biotech Company, Tianjin, China) as described previously [29]. Overnight cultures of *K. pneumoniae* were adjusted to 1×10^8^ colony forming units (CFU) / mL using phosphate-buffered saline. *G. mellonella* larvae were injected with 10 μL of the bacterial culture and incubated in the dark at 37°C for 5 days; survival was continuously monitored. *K. pneumoniae* NTUH-K2044 and *K. pneumoniae* ATCC 700603 were used as high and low virulence control strains, respectively. Experiments were carried out in triplicate.

### Antimicrobial susceptibility testing

The broth microdilution test was used to determine the minimum inhibitory concentrations (MICs) of CAZ, FEP, IPM, MEM, CZA, ATM, TZP, NIT, AMK, LVX, TGC, and PB in *K. pneumoniae* isolates, which were all purchased from Hangzhou Kangtai Biotechnology Co. The Clinical and Laboratory Standards Institute’s (2020) guidelines were used to interpret the susceptibility breakpoints [30]. The MICs of tigecycline were described following the breakpoint established by the US Food and Drug Administration. *E. coli* ATCC 25922 was used as a quality control strain.

### Detection of capsular serotypes

The genomic DNA of all *K. pneumoniae* strains was extracted from overnight cultured strains using a boiling method [31]. The polymerase chain reaction (PCR) was performed to detect capsular serotype genes (K1, K2, K5, K20, K54, and K57) with primers as previously described [32]. Positive PCR products were subjected to direct Sanger sequencing.

### Biofilm formation assay

The crystal violet staining method was used to test biofilm formation in KP isolates, as described previously [33]. The absorbance at 570 nm was calculated, and data are presented as the mean ± standard deviation for each triplicate assay. We used *K. pneumoniae* ATCC 700606 as a negative control. The OD cut-off (ODc) value was determined using the formula, in accordance with the parameters defined by Ragupathi et al. [34], as follows: ODc = average OD of the negative control + (3 × standard deviation of negative control). The biofilm formation ability was categorized based on optical density results as follows: (1) strong biofilm producer (OD > 4 × ODc); (2) medium biofilm producer (4 × ODc ≥ OD > 2 × ODc); (3) weak biofilm producer (2 × ODc ≥ OD > ODc); and (4) non-biofilm producer (OD ≤ ODc).

### Whole-genome sequencing (WGS)

WGS was used to identify resistant genes and virulence factors in hvKP isolates. Approximately 10 μg of DNA was extracted from each strain using the DNeasy UltraClean Microbial Kit (QIAGEN, Hilden, Germany) to establish two Illumina paired-end libraries with 500 and 2000 base pairs (average insertion lengths).

The following reads were excluded from the raw data: 1) reads with undefined bases of 5 bp, 2) reads with low-quality (≤ Q20) bases of 20 bp, 3) contamination of adapter, and 4) duplicate reads. SOAPdenovo v1.05. was used to assemble the final cleaned reads with genome coverage for each strain × 100 approximately. The CGE server (https://cge.cbs.dtu.dk) was used to identify the resistant genes. The phylogenetic tree was constructed using the BacWGSTdb server using the core genome multilocus sequence typing (cgMLST) and single nucleotide polymorphism (SNP) strategy, as defined previously [35]. The Oxford scheme was used for multilocus sequence typing (MLST), and the sequence types (STs) were allocated through the MLST database (https://bigsdb.pasteur.fr/klebsiella/klebsiella.html). The sequence data were deposited in NCBI with the accession PRJNA745055.

### Statistical analyses

Clinical characteristics, antimicrobial susceptibility, virulence factors, and biofilm formation were compared between the cKP group and the hvKP group. For categorical variables, the *χ*^*2*^ test or Fisher’s exact test was used, and for continuous variables, the Student’s t test was used. SPSS 21.0 was used for statistical analysis, and *P* < 0.05 was considered statistically significant.

## Data Availability

The datasets generated and analyzed during the current study are available from the corresponding author on reasonable request. WGS data appear to have been deposited in NCBI with the accession PRJNA745055.

## Abbreviations

ST: Sequence type
hvKP: Hypervirulent *K. pneumoniae*
KP: *K. pneumoniae*
cKP: Classical *K. pneumoniae*
WBC: White blood cell
MALDI-TOF MS: Matrix-assisted laser desorption/ionization time-of-flight mass spectrometry
CFU: Colony forming units
MIC: Minimum inhibitory concentrations
CAZ: Ceftazidime
FEP: Cefepime
IPM: Imipenem
MEM: Meropenem
CZA: Ceftazidime / avibactam
ATM: Aztreonam
TZP: Piperacillin / tazobactam
NIT: Nitrofurantoin
AMK: Amikacin
LVX: Levofloxacin
TGC: Tigecycline
PB: Polymyxin
PCR: Polymerase chain reaction
OD: Optical density
cgMLST: Core genome multilocus sequence typing
SNP: Single nucleotide polymorphism
MLST: Multilocus sequence typing
WGS: Whole-genome sequencing
UTIs: Urinary tract infections
UC: Urine culture
ASB: Asymptomatic bacteriuria
HCCA: α-cyano-4-hydroxy-cinnamic acid
